# Two-year changes in quality of life in elderly patients with low-energy hip fractures. A case-control study

**DOI:** 10.1186/1471-2474-11-226

**Published:** 2010-09-29

**Authors:** Gudrun Rohde, Glenn Haugeberg, Anne Marit Mengshoel, Torbjorn Moum, Astrid K Wahl

**Affiliations:** 1 Department of Rheumatology, Sorlandet Hospital, Kristiansand, Servicebox 416, 4604 Kristiansand, Norway; 2 Faculty of Health and Sport, University of Agder, Servicebox 422, 4604 Kristiansand, Norway; 3 Institute of Health and Society Medical Faculty, University of Oslo, Pb.1153 Blindern, 0316 Oslo, Norway; 4 Dept. of Behavioural Sciences in Medicine, Medical Faculty, University of Oslo 1111, Blindern, 0317 Oslo, Norway

## Abstract

**Background:**

The long-term effect of hip fracture on health-related quality of life (HRQOL) and global quality of life (GQOL) has not been thoroughly studied in prospective case-control studies.

**Aims:**

a) to explore whether patients with low-energy hip fracture regain their pre-fracture levels in HRQOL and GQOL compared with changes in age- and sex-matched controls over a two year period; b) to identify predictors of changes in HRQOL and GQOL after two years.

**Methods:**

We examined 61 patients (mean age = 74 years, *SD *= 10) and 61 matched controls (mean age = 73 years, *SD *= 8). The Short Form 36 assessed HRQOL and the Quality of Life Scale assessed GQOL. Paired samples *t *tests and multiple linear regression analyses were applied.

**Results:**

HRQOL decreased significantly between baseline and one-year follow-up in patients with hip fractures, within all the SF-36 domains (*p *< 0.04), except for social functioning (*p *= 0.091). There were no significant decreases within the SF-36 domains in the controls. Significantly decreased GQOL scores (*p *< 0.001) were observed both within patients and within controls between baseline and one-year follow-up. The same pattern persisted between baseline and two-year follow-up, except for the HRQOL domain mental health (*p *= 0.193). The patients with hip fractures did not regain their HRQOL and GQOL. Worsened physical health after two years was predicted by being a patient with hip fracture (B = -5.8, *p *< 0.001) and old age (B = -1.0, *p *= 0.015), while worsened mental health was predicted by co-morbidity (B = -2.2, *p *= 0.029). No significant predictors of differential changes in GQOL were identified.

**Conclusion:**

A hip fracture has a long-term impact on HRQOL and is a strong predictor of worsened physical health. Our data emphasize the importance of preventing hip fracture in the elderly to maintain physical health. This knowledge should be included in decision-making and health care plans.

## Background

With age, the incidence of hip fractures in the elderly increases exponentially due to increased prevalence of osteoporosis and increased risk of falls [1-4]. In the elderly, the majority of hip fractures are a result of a low-energy trauma [4,5], defined as falls from standing height or less [6]. Fracture at the hip may imply pain and reduced physical functioning months and years after the fracture [7-9], and may consequently affect quality of life (QOL).

QOL is considered a subjective phenomenon, which is often assessed through self-report and thereby supplements objective factors associated with disease, in this context hip fracture and bone mineral density (BMD). Traditionally, QOL comprises both health-related quality of life (HRQOL) and global quality of life (GQOL) [10]. HRQOL may be defined as the individual's experience of their general state of health, such as physical, social, and mental well-being [11], and GQOL may be defined as a broad range of human experiences related to one's overall well-being and satisfaction, and has a meaning beyond an individual's health [12-14]. A broad QOL perspective includes both the health aspect (HRQOL) and satisfaction with life as a whole (GQOL).

Previous QOL studies in patients with hip fracture have shown that HRQOL decreases *after a hip fracture*, and that physical health is influenced more by the fracture than mental health [8,9,15-18]. Furthermore, HRQOL seems to decrease more after a hip fracture in patients who had low HRQOL before the fracture than in those with higher scores [18]. Additionally, studies focusing on *predictors *of changes in HRQOL after a hip fracture show that low body mass index (BMI) and low BMD are associated with reduced HRQOL two years after a low-energy hip fracture in postmenopausal women [7].

To the best of our knowledge, no studies have assessed whether patients with hip fractures regain their pre-fracture GQOL after the fracture, nor have predictors of changes in GQOL in patients with low-energy hip fracture been identified. Furthermore, the complexity of the consequences of hip fractures, including both an objective perspective (e.g., BMD, BMI, demographic, and clinical indicators) and a subjective perspective (e.g., QOL) on changes, needs to be addressed. Knowledge about these issues may provide patients, providers, and decision makers with important information on the impact of disease and treatment on physical, psychological, social functioning, and well-being, and satisfaction with life. Thus, the patient's subjective perspective may be included in decision making and health care plans, such as contact and collaboration of health care between the hospitals and the health care units in the municipalities after the fracture [19,20].

Hence, the present study aims to:

a) Explore whether patients with low-energy hip fracture regain their pre-fracture levels in HRQOL and GQOL compared with changes in age- and sex-matched controls over a two year period;

b) Identify predictors of changes in HRQOL and GQOL after two years.

## Methods

### Study design and study population

A case-control design with a prospective follow-up was applied. The study was approved by the Regional Committee for Medical Research Ethics and the National Data Inspectorate.

Patients with low-energy hip fractures aged 50 years and older were recruited from an osteoporosis centre at a regional hospital in southern Norway in 2004 and 2005. The hospital is the only referral centre for orthopaedic trauma in the region. Patients assessed at the osteoporosis centre and willing to participate in a study with assessment at the time of fracture and at one- and two-year follow-up were included. Before inclusion, we made sure that the fracture was not a result of high-energy trauma and was caused only by minimal trauma according to the definition of low-energy fracture [6]. We excluded patients with confusion or dementia, serious infection, patients not capable of giving informed consent, patients not capable of speaking Norwegian, and tourists. A nurse or doctor assessed the cognitive function of the patients using a clinical judgment. Four trained osteoporosis - nurses recruited the patients and the controls, and administered the clinical assessments and the questionnaires.

**Figure 1:**
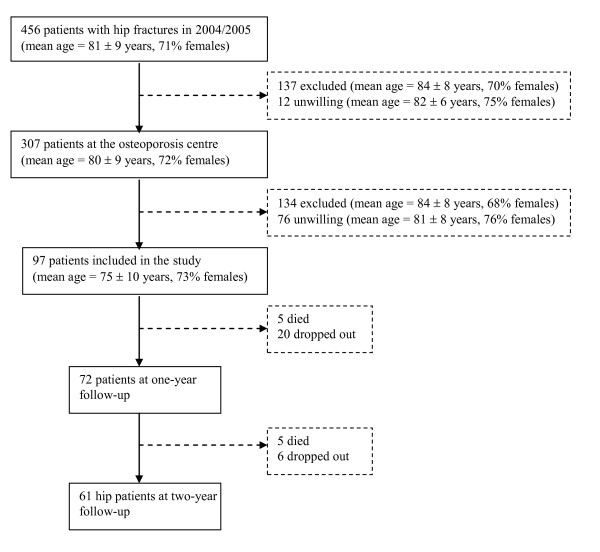
**Flow chart of the patients with hip fractures at baseline and at one- and two-year follow-up**.

During the two-year inclusion period, 456 patients with a low-energy hip fracture were treated at the hospital. Three hundred and seven of the patients were examined clinically at the osteoporosis centre, and 97 patients were included in the study (Fig. 1). Of the patients with hip fracture who were excluded from the examination at the osteoporosis centre or from participating in the study, most were excluded because of dementia or because they were unable to give informed consent.

Two hundred and twenty-six age- and sex-matched controls were randomly allocated from the national registry for the catchment area and invited by mail to participate in the study at baseline. The controls were identified consecutively along with patient recruitment. If a potential control refused to participate or did not respond to the invitation, a new control was invited. Potential controls with a fracture the year before inclusion, dementia or not capable of speaking Norwegian were excluded. At two year follow-up we sex- and age-matched (± five years) the 61 patients with a hip fracture who were still in the study with 61 controls who had valid measures at baseline and at one- and two-year follow-up.

### Data collection procedure

Patients were assessed at the osteoporosis centre and included in the study a median of four days after the fracture (interquartile range = two days). BMD measurements were obtained and the patients were asked about the time before the fracture occurred with regard to demographic and clinical variables. Furthermore, exercise, falls, and the use of health care resources the year before the fracture were assessed. The controls were asked these questions with reference to the period immediately preceding inclusion. The patients were asked to recall their HRQOL (Short Form 36) the four weeks before fracture and GQOL (Quality of Life Scale) at the time preceding the fracture, and the controls with reference to the weeks and time preceding inclusion. These data were used as baseline measures for both groups. The same data collection performed at baseline was repeated after one and two years.

### Instruments at baseline and at one- and two-year follow-up

#### Demographic and clinical variables

Demographic data, height and weight (to calculate BMI), whether the patients and controls exercised for at least 30 minutes three times a week (walking or more intensive exercise), self-reported co-morbidity, including heart diseases, pulmonary diseases, neurological disorders, urogenital disorders, gastrointestinal disorders, endocrine disorders, inflammatory joint disorders, connective tissue disorders, cancer, and mental disorders, medication, smoking habits, and the number of self-reported falls (without counting the fall related to the fracture) the year before the fracture (inclusion in the controls) were recorded. For co-morbidity, we also computed a sum score of the number of diseases in each patient, which was used in the multivariate analyses.

#### Bone density measurements

Standardized BMD measurements at lumbar spine L2-4 and femoral-neck and total hip on the non-fractured hip (both hips in the controls) were performed by four trained nurses using the same dual energy x-ray absorptiometry (DXA) equipment (General Electric, Lunar Prodigy) at baseline and at one- and two-year follow-up. The machine was stable over the entire measurement period. Long-term spine phantom in vitro coefficient of variation (CV) for the whole period was 0.62%. The in vivo CV for the measurement procedure was 1.19% at lumbar spine L2-4, 0.95% at right total hip, and 0.89% at left total hip. The BMD measurements were expressed as T-scores (*SD*) with calculations based on the reference values in the DXA machine provided by the manufacturer. Osteoporosis was defined as T-score ≤ -2.5, osteopenia as T-score > -2.5 and < -1.0, and normal BMD as T-score ≥ -1.0, according to the WHO definition for osteoporosis [6].

#### HRQOL: Short Form-36 (SF-36)

The SF-36 was used to assess HRQOL [21,22]. The questionnaire consists of 36 questions of self-reported aspects of health. The SF-36 comprises eight domains: physical functioning, physical role limitations, bodily pain, general health, vitality, social functioning, emotional role limitations and mental health, which also were combined into physical and mental component summary scales (PCS and MCS). Norwegian SF-36 norms were used to calculate the summary scales [23]. PCS and MCS indicate self-reported physical and mental health, respectively. The SF-36 scales were scored according to published scoring procedures, and each scale was expressed using values from 0 to 100, with 100 representing excellent health [21,22]. At baseline seven of the patients and 10 of the controls had one or more missing responses. At one year follow-up 10 of the patients and 15 of the controls had one or more missing responses, and at two year follow-up 14 of the patients and 12 of the controls had one or more missing responses. Imputations for missing responses were carried out in accordance with the guidelines given by the developers of the questionnaire. Results from earlier studies indicate satisfactory reliability and validity of the SF-36 [21,22,24,25]. For the entire study population, Cronbach's alphas in the study in the eight SF-36 domains at baseline were 0.91 for physical function, 0.86 for physical role limitation, 0.86 for bodily pain, 0.70 for general health, 0.87 for vitality, 0.88 for social function, 0.75 for emotional role limitation and 0.78 for mental health.

#### GQOL: Quality of Life Scale (QOLS)

The QOLS is a 16-item self-report instrument that measures GQOL [14,26,27]. In the questionnaire, GQOL is understood as a broad range of human experiences related to one's overall well-being and satisfaction, and comprises relationship and marital well-being, health and functioning, and personal, social, and community commitment [12,26,28]. The items are rated on seven-point satisfaction scales. The questionnaire is scored by adding up the items to obtain a total score from a minimum of 16 to a maximum of 112. Higher scores indicate better GQOL. At baseline 25 of the patients and 17 of the controls had one or more missing responses. At one year follow-up 32 of the patients and 26 of the controls had one or more missing responses, and at two year follow-up 27 of the patients and 30 of the controls had one or more missing responses. The items with most missing responses were QOLS item number four (having and rearing children) and item five (close relationship with spouse or other significant other). For incomplete questionnaires, the missing values were replaced with the mean value of the answered items of the respondent when at least 80% of the items had a valid response. Results from earlier studies show satisfactory reliability and validity of the questionnaire [27,29-31]. For the entire study population, Cronbach's alpha in the QOLS at baseline was 0.83.

### Statistical analyses

Statistical analyses were carried out using the Statistical Package for Social Sciences (SPSS) for Windows (version 16.0). Chi-square tests and independent *t *tests were used to compare differences between groups. Paired samples *t *tests were used to compare HRQOL and GQOL between baseline and one-year follow-up, and between baseline and two-year follow-up, in patients and controls. General linear model (GLM) (repeated MANOVA) were also applied to examine differences in the repeated HRQOL and GQOL measures within the groups. Furthermore, standard difference scores (s-scores) were calculated by subtracting the mean SF-36 or QOLS scores at baseline from the mean scores of the one- and two-year follow-ups, and then dividing by the *SD *at baseline within the groups [32]. The s-scores allow for comparisons across dependent variables and were interpreted according to Cohen's effect size index, with 0.2 indicating a small difference, 0.5 a moderate difference, and 0.8 or more a large difference [33,34]. Independent sample *t *tests were used to compare differences in HRQOL and GQOL between patients and controls at baseline, one- and two-year follow-up.

Multiple linear regression analysis (procedure GLM in the SPSS) was used to identify significant predictors of changes in HRQOL (delta PCS and delta MCS) and GQOL (delta QOLS) in the study-population (both patients and controls). Delta scores (change scores) were calculated by subtracting the baseline PCS, MCS and QOLS scores from the two year PCS, MCS and QOLS follow-up scores, respectively. Independent variables in the multiple regression analyses were the demographic variables of age (in five-year groups), sex, and marital status (cohabiting/living alone), and the clinical variables of BMD (normal BMD/. osteopenia/osteoporosis), patients/controls, and co-morbidity (a sum score of the number of diseases). These variables have been shown to be covariates of HRQOL and/or GQOL in earlier studies [33]. The regression analyses were adjusted for total PCS, MCS, or QOLS, respectively, at baseline. To explore how much of the explained variance in HRQOL and GQOL decline was uniquely attributable to fracture, the patients/controls variable was excluded from the multiple regression analyses while retaining the other predictors in the equation. To test if the independent variables in the regression models (potential effects predictors) of change in our dependent variables were significantly different for patients and controls, interaction terms involving the patient/control dichotomy and each of the independent variables were entered one pair at a time, while retaining the other independent variables (main effects) in the model. The level of significance was set at 0.05.

## Results

### Demographic and clinical characteristics

#### Baseline

Demographic and clinical characteristics at baseline of those participants who completed the two-year follow-up assessments are shown in Table 1. Compared with the controls, the patients with hip fractures had significantly lower weight (*p *< 0.001), lower BMI (*p *< 0.001), were more likely to have osteoporosis (*p *< 0.001), live alone (*p *= 0.045), be current smokers (*p *= 0.019), and exercise less (*p *= 0.033). Two years after baseline, the same differences between the groups were present, and, in addition, the patients with hip fractures more often used antiresorptive treatment (ART) than controls, 35 (57%) vs 11 (18%) (*p *< 0.001).

**Table 1:** Demographics and clinical characteristics in patients with hip fractures (n = 61) and controls (n = 61) at baseline.

	Patients with hip fractures		Controls		p*-value
	Mean (*SD*)	n (%)	Mean (*SD*)	n (%)	
**Demographics**					
Age (years)	73.7 (9.5)		72.6 (8.2)		0.502
(range 50-90 years)					
Females		46 (75)		46 (75)	1.000
Current height (cm)	167.8 (8.7)		166.8 (7.9)		0.513
Current weight (kg)	66.4 (12.2)		74.7 (12.5)		**< 0.001**
BMI (kg/m^2^)	23.6 (3.7)		26.8 (3.7)		**< 0.001**
Education					0.525
< 10 years		26 (46)		22 (37)	
11-13 years		18 (32)		21 (35)	
> 13 years		12 (21)		17 (28)	
Cohabiting		26 (44)		38 (62)	**0.045**
Regular exercise**		36 (59)		47 (77)	**0.033**
Current smoker		16 (36)		6 (10)	**0.019**
**Clinical characteristics**					
Current calcium and/or vitamin D treatment		12 (20)		18 (30)	0.207
Current ART		9 (15)		10 (16)	0.803
Current glucocorticoid treatment		9 (16)		3 (5)	0.083
Previous fractures		28 (46)		29 (48)	0.362
Mother fracture		18 (30)		18 (30)	0.930
≥ one fall in the previous year		27 (47)		19 (37)	0.289
Osteoporosis ***		35 (58)		12 (20)	**0.001**
Osteopenia		21 (34)		29 (48)	
Normal BMD		5 (8)		19 (32)	
Heart diseases		28 (48)		30 (49)	0.856
Pulmonary diseases		10 (16)		5 (8)	0.168
Neurological diseases		8 (13)		3 (5)	0.114
Endocrine disorders		5 (8)		4 (7)	0.729
Gastrointestinal disorders		6 (10)		11 (18)	0.191
Urogenital disorders		5 (8)		2 (3)	0.243
Inflammatory joint/connective tissue disorders		16 (26)		13 (21)	0.523
Cancer		8 (13)		6 (10)	0.570
Mental disorders		3 (5)		2 (3)	0.648
Co-morbidities (range 0-6) ****	1.2 (1.1)		1.5 (0.9)		0.218

Continuous variables are presented as mean (*SD*) and group variables as numbers (%).

* Chi-square when comparing categorical data. Independent *t *tests with continuous variables. Bold *p*-values indicate significant differences between the groups. ** Exercise more than 30 minutes three times a week.

*** Osteoporosis at total hip and/or spine L2-L4.

**** Mean (SD) score of diseases including: heart diseases, pulmonary diseases, neurological disorders, urogenital disorders, gastrointestinal disorders, endocrine disorders, inflammatory joint disorders and connective tissue disorders, cancer and mental disorders.

BMI, body mass index; ART, antiresorptive treatment (specific osteoporosis treatment comprising biphosphonates, or selective oestrogen-receptor modulators).

Only limited information was available with regard to the patients excluded from examination at the osteoporosis centre (n = 137) or from participating in the study (n = 134), and the patients unwilling to visit the osteoporosis centre (n = 12) or unwilling to participate in the study (n = 76) (Fig. 1). Of the patients excluded from examination at the osteoporosis centre, 76 died during the two-year period after the fracture, while of the patients unwilling to visit the osteoporosis centre, two patients died during the two-year period after the fracture. Of the patients examined at the osteoporosis centre but excluded from the study, 47 died during the two-year period after the fracture, while of the patients unwilling to participate in the study, 16 patients died during the two-year period. Of the 456 patients with hip fracture 33% died during a two-year period after the fracture.

#### Dropouts

Of the 36 patients who dropped out during the two-year follow-up period of the study, five died during the first year of follow-up and another five died during the second year of follow-up (Fig. 1). No controls died during the study period. There were no significant differences in age at baseline between the patients who attended the two-year follow-up and the 10 patients who died or the 26 patients who dropped out for other reasons. Baseline scores of the 36 patients who dropped out showed that more of these patients were living alone (*p *= 0.031), and reported endocrine disorders (*p *= 0.023), as well as lower scores within the HRQOL sub-dimensions general health (p = 0.031) and vitality (p = 0.009).

### Changes in HRQOL and GQOL

#### Baseline compared with one year after fracture

**Figure 2:**
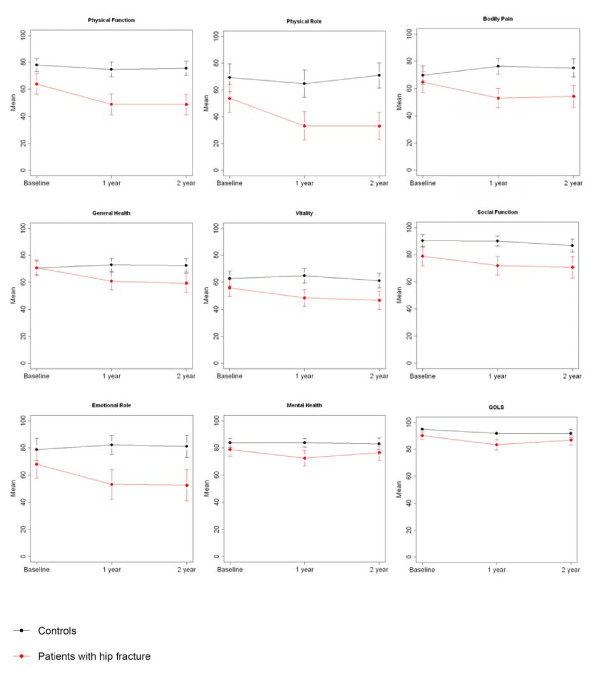
**HRQOL and GQOL mean scores (95% confidence intervals) at baseline, and at one- and two-year follow-up in patients with hip fractures (n = 61) and controls (n = 61)**.

**Figure 3:**
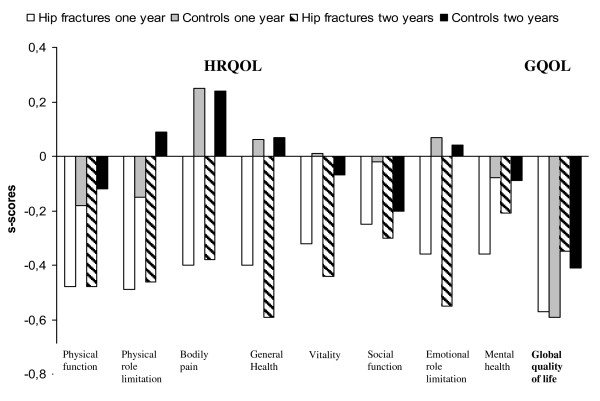
**Standard difference scores (s-scores) of HRQOL and GQOL at one- and two-year follow-up, compared with baseline for patients with hip fracture (n = 61) and controls (n = 61) with valid HRQOL and GQOL change-scores**.

HRQOL decreased significantly between baseline and one-year follow-up in the patients with hip fractures, within all SF-36 domains (including PCS and MCS) (*p *< 0.04), except for social functioning (*p *= 0.091). The decrease within the SF-36 domains persisted after using GLM analyses (Fig. 2). The highest s-scores were observed within physical functioning (s-score = -0.48), and physical role limitations (s-score = -0.49) (Fig. 3). There were no significant decreases in the SF-36 scores in the controls (Fig. 2). Significantly decreased GQOL scores (*p *< 0.001) were observed both in the patients with hip fractures and in controls at one-year follow-up (Fig. 2), with s-score = -0.57 and s-score = -0.59 respectively (Fig. 3).

#### Baseline compared with two years after fracture

Compared with baseline, patients with hip fractures reported significantly decreased HRQOL scores within all SF-36 domains (including PCS and MCS) (*p *< 0.03), except for social functioning (*p *= 0.073) and mental health (*p *= 0.193). The decrease within the SF-36 domains persisted after using GLM analyses. Moderate s-scores were observed within general health (s-score = -0.59) and emotional role limitations (s-score = -0.55). Physical functioning, and physical role limitations showed s-score = -0.48 and s-score = -0.46, respectively. There were no significant decreases in the controls (Fig. 3). Furthermore, significantly decreased GQOL scores (*p *< 0.05) were observed both in the patients with hip fractures and in controls at two-year follow-up (Fig. 2), with s-scores = -0.35 and s-score = - 0.41, respectively (Fig. 3).

#### Differences between patients with hip fractures and controls

At baseline, i.e. prior to the fracture, the patients with hip fractures reported significantly lower scores in the HRQOL (SF-36) domains of physical functioning (*p *= 0.003), physical role limitation (*p *= 0.038), social functioning (*p *= 0.009), and in GQOL (QOLS) (*p *= 0.013). At one year follow-up, the patients reported lower HRQOL in all of the eight domains and in GQOL (*p *< 0.01) compared to controls. The same pattern persisted at two-year follow-up, except for the HRQOL domain mental health (*p *= 0.08). The differences in the PCS scores between patients (mean (SD)) 47 (11) and controls 50 (8) (*p *= 0.094), and in the MCS scores, 49 (11) vs 52 (8) respectively, (*p *= 0.109) at baseline were not significant. However, at one year follow-up there were significant differences between the patients in the PCS scores 42 (11) and controls 51 (8), (*p *< 0.001), and in the MCS scores 46 (12) and 52 (7), (*p *= 0.002). The same pattern persisted at two-year follow-up showing a PCS score in the patients of 42 (11) and in the controls 51 (8), (*p *< 0.001), and MCS scores of 46 (13) and 51 (8), (*p *= 0.037).

### Predictors of change in HRQOL and GQOL at two-year follow-up

Worsened physical health (SF-36-PCS) at two-year follow-up was predicted by being a patient with hip fracture (B = -5.8, *p *< 0.001) and old age (B = -1.0, *p *= 0.015). The variance in change uniquely attributable to fracture was 0.267-0.156 = 0.111 (= 11%). Worsened mental health (SF-36-MCS) was predicted by co-morbidity (B = -2.2, *p *= 0.029), and the variance in change uniquely attributable to fracture was 0.215-0.195 = 0.02 = 2%) (Table 2). Interaction terms between pairs of each independent variable and patients/controls (tested one pair at a time) revealed a significantly stronger effect of old age on changes of physical health (PCS) among patients with hip fracture than among controls. Furthermore, the negative effect of multiple co-morbidities at baseline was particularly pronounced on changes of mental health (MCS) among the patients.

**Table 2:** Predictors of change in HRQOL (delta PCS and delta MCS) and GQOL (delta QOLS) in patients with hip fractures (n = 61) and controls (n = 61).

	PCS			MCS			QOLS		
	**Adj B**	**95% CI**	***p***	**Adj B**	**95% CI**	***p***	**Adj B**	**95% CI**	***p***

**Demographic**									
Age*	-1.0	(-1.9, -0.2)	**0.015**	0.05	(-1.1, 1.2)	0.926	-0.4	(-1.7, 0.9)	0.529
Male	-0.5	(-3.8, 2.9)	0.783	-2.4	(-7.0, 2.2)	0.310	-1.4	(-6.5, 3.7)	0.590
Female	Ref			Ref			Ref		
Living alone	-2.9	(-5.8, 0.0)	0.050	3.5	(-0.6, 7.5)	0.093	-0.1	(-4.6, 4.4)	0.968
Cohabiting	Ref			Ref			Ref		
**Clinical**									
Hip patients	-5.8	(-8.7, -2.8)	**< 0.001**	-3.8	(-7.9, 0.3)	0.071	-1.7	(-6.1, 2.7)	0.447
Controls	Ref			Ref			Ref		
Osteopenia**	-0.8	(-4.3, 2.8)	0.671	-0.5	(-5.5, 4.5)	0.837	-3.2	(-8.8, 2.4)	0.265
Osteoporosis**	0.2	(-3.9, 4.3)	0.918	-2.1	(-7.9, 3.6)	0.461	1.4	(-4.7, 7.6)	0.649
Normal BMD	Ref			Ref			Ref		
Co-morbidity	0.0	(-1.4, 1.4)	0.999	-2.2	(-4.1, -0.2)	**0.029**	-0.3	(-2.6, 2.0)	0.783
**QOL**									
PCS incl.	-0.3	(-0.5, -0.2)	**< 0.001**						
MCS incl.				-0.5	(-0.8, -0.3)	**< 0.001**			
QOLS incl.							-0.2	(-0.5, 0.0)	**0.049**
**R_2 _adj**	26.7%			21.5%			2.3%		

Adjusted unstandardized regression coefficients, 95% CI, and *p *values.

*p*-values marked with bold indicate statistically significance. The demographic and clinical variables in column one are used in all three

models, in addition PCS at inclusion is used in model one, MCS at inclusion in model two and QOLS at inclusion in model three

* Age in five-year groups.

** Osteopenia/osteoporosis at total hip and/or spine L2-L4.

BMD, bone mineral density; PCS, SF-36 physical component summary scale; MCS, SF-36 mental component summary scale

(range 0-100, where 100 = high HRQOL); QOLS, Quality of Life Scale (16-112, where 112 = high GQOL).

We found no significant predictors of differential change in GQOL, and no variance in change was uniquely attributable to fracture (Table 2).

## Discussion

Compared with pre-fracture the patients with hip fractures reported modestly or moderately decreased HRQOL and GQOL one year after fracture that remained nearly unchanged at two-year follow-up, whereas the controls reported no significant changes in HRQOL and moderately or modestly decreased GQOL. Changes in HRQOL physical health two years after baseline were predicted by age and being a patient with hip fracture, while co-morbidity predicted changes in HRQOL mental health.

Our findings of lower pre-fracture scores of patients compared with controls within some HRQOL domains and decreased HRQOL one year after a low-energy hip fracture, especially within the physical domains, are in line with earlier studies in the field [8,9,17,18,35]. Furthermore, the patients seem to reach a plateau with regard to HRQOL one year after the fracture. Our findings show that the burden of a hip fracture on self-reported physical health is particularly pronounced among the oldest patients. These findings might emphasize that health care providers should have a special focus on the oldest patients with regard to targeted rehabilitation efforts, so that the patients could achieve the highest possible function and independence level in everyday life.

The patients with hip fractures had lower GQOL before the fracture occurred, compared with controls. However, both the patients with a hip fracture and the controls reported moderately decreased GQOL one year after inclusion and modestly decreased GQOL two years after inclusion. We might interpret these changes as being to aging. The findings are in line with changes over a period of one or two years in other patient groups [36,37]. In contrast to our results, studies have shown that GQOL does not seem to be influenced by old age [38,39]. Furthermore, it is possible that those patients and controls who agreed to participate in our study did so at a point in time when their GQOL was better than their own typical (long-term) level, thus creating a "regression to mean" effect two years later when these same individuals may have returned to their usual level of GQOL [33]. There seems to be a difference regarding how a low-energy hip fracture influences GQOL and HRQOL over a period of two years, as the patients' experiences of change in overall satisfaction with life (i.e., GQOL) appear to be in line with that of the elderly population in general. In contrast, the patients' experience of their health (i.e., HRQOL) is substantially influenced by the fracture.

To be a patient with a hip fracture was a strong predictor of worsened physical health in an elderly population, even when known correlates of decreased physical health such as co-morbidity, age, and marital status [40-42] were adjusted for. This indicates a strong association between a hip fracture and worsened health. In contrast to previous studies, we did not find BMD as a significant predictor of changes in HRQOL, and there is no clear explanation for this [8,16-18,35,43]. The findings underline the burdens and complexity related to a hip fracture.

The overview of the excluded patients and the patients unwilling to participate in this study shows that those who were included were probably the healthiest and the youngest ones. The majority of the excluded patients were excluded because of dementia or because they were unable to give informed consent. Furthermore, nursing home patients who were sent home within two days after fracture were not included in the study. These patients suffered from mental and physical diseases, and were less able to take care of activities of daily living than the patients included in the study. However, even in the relatively exclusive group of patients included in the study, the baseline HRQOL level was not regained over the two-year period. Furthermore, the relatively high number of excluded patients indicates that many patients with hip fractures are not capable of self-reporting their HRQOL and GQOL. This may be the reason why patient-based outcomes such as HRQOL and GQOL among patients with hip fractures are available only for rather limited samples. Thus, the knowledge derived from this study, as well as others, can probably be generalized primarily to a rather healthy sub-group of the elderly population with hip fractures. We do not know the reason for the 10 deaths among the patients during the two-year follow-up. However, we could assume that the deaths might be related to age, co-morbidity or complications associated with the hip fracture.

The patients' ability to correctly recall their HRQOL and GQOL before the hip fracture may be questioned. One possible way is to apply epidemiological surveys of QOL and from a huge cohort identify people who fracture, and thereafter examine how they manage compared with those not fracturing. To perform such a large study was not possible for us to do. And a cohort study would also have limitations because QOL may change between the time of data capture and fracture, and thus may no longer be valid for the time of fracture. Thus, an alternative method is to use pre-injury recall, like in this trauma study and in other studies [9,18,35]. Changes in health status, such as that resulting from experiencing a fracture, might cause a shift in how the patients judged their HRQOL and GQOL (selective reporting bias and response shift) [44]. On the other hand, patients who have experienced a recent change in health are more likely to make accurate health-related responses [32,45,46]. To minimize the recall problem it is recommended that QOL assessments should be performed with the shortest possible time lag after the fracture event, which we aimed for in our study. The elapsed time from fracture to assessment was relatively short and most patients completed the baseline QOL questionnaires within five days after fracture. Thus, it seems unlikely that the patients would have forgotten about their QOL before and at the time of the fracture. To minimize further the retrospective design of our baseline data, the questionnaires were administered with an instruction that the patients should think of the period before the fracture. Furthermore, demographic and clinical characteristics before the fracture were also based on recall, and the validity on reports of numbers of e.g. fall may be questioned. Two studies [47,48] have shown that the number of falls reported by recall, may be underestimated among elderly. However, as the participants are the only source of information in these matters, we are forced to rely on self-reports.

This study has a case-control, prospective, and longitudinal design, and population based controls were chosen as the control population. We could argue that a hospital based control population would have been better to assess the influence of fracture as well as the outcome. On the other hand, the disease giving rise to the hospitalization and the impact of the disease, would have influenced characteristics and outcomes in ways which would be hard to assess. Furthermore, comparisons with elderly from the general population were a guiding principle in this study. Other studies have recruited controls from general practitioners [8,18], both cases and controls were recruited from general practitioners [17,43,49], or population-based HRQOL (SF-36) norms were used in cross-sectional comparisons [7,16,35].

A low-energy hip fracture seems to have substantial clinical implications with respect to HRQOL in most patients. Elderly people who barely manage alone before the fracture might be in need of assistance afterwards. This assistance might include both practical help and community care. An intensive interdisciplinary approach is therefore required to improve functioning in everyday life, which would also include a focus on patient-reported outcomes such as HRQOL and GQOL. Furthermore, the impact of a hip fracture on self-reported outcomes, such as the HRQOL revealed in our study, might give support to the implementation of patient-reported outcomes in daily clinical practice, and thereby reach the patient's perspective and evaluation of the health care both in the hospitals and the rehabilitation units in the municipalities [19,20]. With a growing number of elderly and an incidence of hip fractures in Norway that is among the highest in the world [2-4], low-energy fractures seem to be a challenge for both the society and the individuals in the years to come. An increased contact and collaboration between the different levels of care might thereby be required, i.e. between hospitals and community health care [19,20].

## Conclusion

The patients with hip fractures reported significantly decreased HRQOL and GQOL one year after the fracture. Furthermore, minor changes in HRQOL and GQOL between one- and two-year follow-up were seen, and the patients did not regain their HRQOL and GQOL. However, the controls also reported decreased GQOL at one- and two-years follow-up. Moreover, a hip fracture is a strong predictor of worsened physical health. The findings of this study may be used to highlight the importance of disease and trauma prevention, early and targeted rehabilitation efforts to increase function and thereby coping after the fracture, and effective contact and collaboration between the hospital and the care unit in the municipality to reach these goals.

## Abbreviations

ART: antiresorptive treatment; BMD: bone mineral density; BMI: body mass index; DXA: dual-energy X-ray absorptiometry; GLM: General Linear Model; GQOL: global quality of life; HRQOL: health-related quality of life; MCS: mental component summary; PCS: physical component summary; SF-36: Short Form-36; s-score: standard difference score; QOLS: Quality of Life Scale; WHO: World Health Organization

## Competing interests

The authors declare that they have no competing interests.

## Authors' contributions

GR initiated this paper as a part of a larger study of fracture patients, collected and analyzed the data and wrote the manuscript. GH was the principal investigator for the study and supervised GR. AM supervised GR during the analyzes and drafting of the paper. TM provided statistical advice. AKW supervised GR during the analyzes and drafting of the paper. All authors critiqued revisions of the paper and approved the final manuscript

## Pre-publication history

The pre-publication history for this paper can be accessed here:

http://www.biomedcentral.com/1471-2474/11/226/prepub
